# Real-time and accurate calibration detection of gout stones based on terahertz and Raman spectroscopy

**DOI:** 10.3389/fbioe.2023.1218927

**Published:** 2023-07-13

**Authors:** Han Li, Yuxin Zhou, Yi Wu, Yanfang Jiang, Hui Bao, Ai Peng, Yongni Shao

**Affiliations:** ^1^ The First Rehabilitation Hospital of Shanghai, School of Medicine, Tongji University, Shanghai, China; ^2^ Shanghai Institute of Intelligent Science and Technology, Tongji University, Shanghai, China; ^3^ Terahertz Technology Innovation Research Institute, Terahertz Spectrum and Imaging Technology Cooperative Innovation Center, Shanghai Key Lab of Modern Optical System, University of Shanghai for Science and Technology, Shanghai, China; ^4^ Shanghai Tenth People’s Hospital, Tongji University School of Medicine, Shanghai, China

**Keywords:** gout stones, spectral techniques, terahertz technique, Raman spectroscopy, handheld instruments

## Abstract

Gout is a metabolic disease that can result in the formation of gout stones. It is essential to promptly identify and confirm the type of gout stone to alleviate pain and inflammation in patients and prevent complications associated with gout stones. Traditional detection methods, such as X-ray, ultrasound, CT scanning, and blood uric acid measurement, have limitations in early diagnosis. Therefore, this article aims to explore the use of micro Raman spectroscopy, Fourier transform infrared spectroscopy, and Terahertz time-domain spectroscopy systems to detect gout stone samples. Through comparative analysis, Terahertz technology and Raman spectroscopy have been found to provide chemical composition and molecular structure information of different wavebands of samples. By combining these two technologies, faster and more comprehensive analysis and characterization of samples can be achieved. In the future, handheld portable integrated testing instruments will be developed to improve the efficiency and accuracy of testing. Furthermore, this article proposes establishing a spectral database of gout stones and urinary stones by combining Raman spectroscopy and Terahertz spectroscopy. This database would provide accurate and comprehensive technical support for the rapid diagnosis of gout in clinical practice.

## 1 Introduction

Gout stone, also known as urate crystal deposition disease, is a metabolic disorder that occurs due to the inability of the body to discharge uric acid normally, resulting in the accumulation of urate crystals in various parts of the body, including joints, soft tissues, kidneys, and the urinary tract. These crystals can trigger inflammatory reactions and cause pain (Parthasarathy and Vivekanandan, 2018). The formation of gout stones is common in joints and surrounding soft tissues such as ligaments and tendons, with the most frequently affected joint being the big toe joint. It can lead to joint inflammation, swelling, and damage, with the potential for joint deformation, distortion, or destruction, particularly in smaller joints such as the toes (Perez-Ruiz et al., 2015). Additionally, gout stones can also develop in various external joint positions and conventional areas, such as the cardiovascular, renal, and spinal regions, leading to systemic diseases, such as urinary tract stones, chronic urate nephropathy, and cardiovascular and cerebrovascular diseases (Ahmad et al., 2021).

Currently, medical imaging techniques such as X-ray, ultrasound, computed tomography (CT), and magnetic resonance imaging (MRI), as well as blood uric acid measurement methods, are commonly used to detect gout stones. X-ray was the first technique used to detect gout stones, as it can display the distribution, shape, quantity, and size of gout stones around joints and soft tissues and identify their connection with bones. However, X-rays cannot detect gout stones smaller than 1 mm or located deep within the tissues, and they cannot detect early gout stone deposits (Fernandes et al., 2017). Ultrasound is a non-invasive detection method that can detect early symptoms of gout, such as swelling and inflammation of soft tissues around joints, and it can detect gout stones smaller than 1 mm. However, the accuracy of an ultrasound examination is affected by hypertrophic soft tissues and cannot detect deep gout stones, and different operators may produce varying results (Kaeley et al., 2020). CT scanning is a high-resolution imaging technique that is more accurate than an X-ray in diagnosing gout stones located in deep tissues and skeletal structures, particularly when X-ray examination is unclear. In addition, CT scanning can also be employed for detecting uric acid deposition of gout stones in the kidneys. However, it has higher costs and radiation compared to other imaging examination methods, which may pose certain health risks to patients and is not suitable for long-term monitoring (Dalbeth et al., 2019). Magnetic resonance imaging (MRI) provides high-resolution images and performs well in detecting small gouty stones within joints. Furthermore, it provides detailed information about joint soft tissues and bones, which can be utilized to diagnose complications of gout. However, MRI examinations require high costs in terms of time and money. Additionally, MRI has limitations, such as the inability of patients with pacemakers, metal implants, or pregnant women to undergo MRI examinations (Davies et al., 2019). In addition to imaging examinations, measuring blood uric acid levels can serve as an auxiliary means for the early diagnosis of gout and monitoring and evaluating gout treatment, but changes in blood uric acid levels are influenced by various factors, such as diet, weight, medication, *etc.*, and therefore have certain limitations (Reginato et al., 2012). However, these methods cannot distinguish gout stones from other types of crystals, and the detection and time costs are relatively high. Different detection method pairs are shown in Table 1.

**TABLE 1:** Comparison of traditional medical detection methods.

Test method	Advantages	Disadvantages	Reference
X-ray	Detects early gout stones	High radiation level, unable to detect gout stones less than 1 mm or located deep, unable to detect early gout stone deposits	Fernandes et al. (2017)
ultrasonic	Detects early symptoms of gout	Highly affected by hypertrophic soft tissue and abdominal gas, unable to detect deep gout stones	Kaeley et al. (2020)
CT scanning	More accurate diagnosis when X-ray examination is unclear	High cost and high radioactivity, not suitable for long-term monitoring	Dalbeth et al. (2019)
MRI	Diagnoseable complications of gout	The testing cost is relatively high, and there are limitations such as patients carrying pacemakers or metal implants, or pregnant women not being able be checked	Davies et al. (2019)
Blood uric acid measurement	Detects the main pathological feature of gout	Blood uric acid levels are influenced by various factors, such as diet, weight, and medication, making it less reliable as a standalone measurement	Reginato et al. (2012)
Near-infrared	Detects physical and chemical properties of gout stones	Low resolution and sensitivity, small structural changes and complex composition may overlook pathological features	Jamrógiewicz (2012)

In recent years, due to its high sensitivity and a non-destructive testing process that is simple and fast, optical technology has been widely used in various types of testing. Compared with traditional detection techniques, the primary focus of optical technology in the detection of gout stones is on analyzing the physical and chemical properties of gout stones. By studying the chemical composition, structure, morphology, and other aspects of gout stones, we aim to understand the etiology and pathogenesis, and develop prevention and treatment plans for gout. Among the various optical technologies, near-infrared technology is the most widely used for detecting the physical and chemical properties of gout stones. Compared to traditional detection techniques, near-infrared spectroscopy can quickly and accurately detect uric acid salts, proteins, and other components in gout stones. Additionally, the composition and chemical structure of the molecule can be analyzed based on its vibration frequency and absorption characteristics (Czarnecki et al., 2015; Sakudo, 2016). However, the resolution and accuracy of near-infrared imaging are relatively low, which may overlook some essential pathological features, especially for the detection of gout stones with complex composition or small structural changes (Jamrógiewicz, 2012). Therefore, researchers have begun exploring the application of other spectral techniques in detecting gout stones. Raman spectroscopy and Terahertz spectroscopy have become two widely studied technologies that can improve the accuracy and precision of gout detection.

Raman spectroscopy is a type of molecular spectroscopy that reflects molecular vibrations and rotations. It characterizes the interaction between molecules and photons, which leads to the characteristic frequency shift of molecular vibrations and rotational energy level differences. The differences in vibration amplitude and rotational energy levels among different substances result in distinct Raman frequency shifts. Raman technology can obtain molecular vibration and chemical structure information of substances by measuring Raman scattering spectral lines in the sample scattering spectrum (Jones et al., 2019). Raman technology has been widely used in analyzing and identifying the characteristic Raman spectral lines of different organic compounds in samples. For example, it has been used to detect organic compounds, such as additives, pigments, and preservatives in food, as well as in water quality testing, environmental monitoring, and medical fields (Auner et al., 2018). Tamosaityte et al. applied Raman spectroscopy technology to detect and analyze different types of urinary stones, and the results showed that Raman technology can accurately identify different types of urinary stones and achieve a quantitative analysis of their chemical components with high accuracy and reliability (Sandra et al., 2022). Zhu et al. (2022) used a portable Raman system to analyze urine stone samples obtained from 300 patients and demonstrated that Raman spectroscopy can be applied to portable automated analysis systems, which have the characteristics of simple operation, easy automation, and rapid detection in on-site clinical environments. Niessink et al. (2023) measured the crystals in joint synovial fluid using Raman spectroscopy, which indicated that Raman technology could be used to evaluate disease conditions or predict the formation of stones. These studies demonstrate that Raman spectroscopy technology provides a fast and convenient method for medical detection.

Terahertz refers to electromagnetic waves with a frequency range between the infrared and microwave regions, specifically ranging from 0.1–10 THz, corresponding to a wavelength range of 30 μm-3 mm (Gowen et al., 2012). Terahertz waves exhibit low photon energy (approximately 0.4–30 meV) and strong penetration, without causing damage or photoionization to the sample. This characteristic enables the use of Terahertz waves for non-contact and non-destructive testing of samples (Guo et al., 2022). Moreover, the Terahertz frequency range covers the intermolecular vibration and rotational energy levels of various organic/biological macromolecules. This ability enables the detection of intermolecular interactions between organic molecules, including hydrogen bonds, van der Waals forces, and others, which are important characteristics of organic molecules in fields such as biology, chemistry, and drug research (Zhang et al., 2022). Jung et al. (2012) employed Terahertz time-domain spectroscopy to detect the distribution of water in human articular cartilage. Their study revealed that Terahertz time-domain spectroscopy technology can non-destructively detect small changes in water content in cartilage, offering a possibility for early diagnosis and treatment of bone and joint diseases. Gilad et al. (2017) utilized Terahertz imaging technology to compare and scan the feet of 30 patients with diabetic foot. The results demonstrated that Terahertz could precisely detect the pathological changes in diabetic foot patients through parameters such as tissue density of the foot and bone density of the foot bottom, and identify possible early pathological changes. This technique provides a new method for real-time and painless detection of the development process of gout foot disease. Zhao Yi employed Terahertz time-domain spectroscopy to detect the components of kidney stones. The study discovered that Terahertz spectroscopy can quickly and accurately detect and differentiate different types of kidney stones (Zhang, 2013; Wu et al., 2016). Additionally, Terahertz time-domain spectroscopy (THz-TDS) can provide spectral information on intramolecular and intermolecular vibrations, which enhances the characterization ability of the substance under test (Shen et al., 2020). In Terahertz spectroscopy experiments, Fourier transform infrared spectroscopy (FTIR) is usually combined to analyze the optical properties of the sample. This is because FTIR technology can achieve high-resolution and high-sensitivity Terahertz spectroscopy measurement in the Terahertz band, and at the same time, time-domain signal measurement can be achieved through Terahertz time-domain spectroscopy technology to analyze and process Terahertz waveforms. These measurement results can be used to determine the physical and chemical properties of the sample, such as the composition, structure, morphology, etc. of the material. Therefore, FTIR technology can serve as a complementary spectral technique to Terahertz time domain spectroscopy system (THz-TDS) and is a suitable tool for studying low-frequency Terahertz spectra of materials. It can provide more comprehensive and in-depth information for Terahertz band analysis and exhibits high accuracy and reliability (Lepodise, 2020; Chamorro-Posada et al., 2016).

Uric acid stones are common in the urinary system and are the only stones that can be dissolved by drugs. It is crucial to have a deep understanding of the pathogenesis of uric acid stones in order to identify, treat, and prevent them effectively. However, many patients with urinary stones are unaware of the properties and causes of their stones, and the composition of stones cannot be determined in hospitals. Therefore, analyzing the components of urinary tract stones is essential for accurate diagnosis and early prevention of gout stones. The extensive use of spectral technology in medical detection has led researchers to recognize its immense potential in disease prevention and detection. In this study, Raman and Terahertz spectroscopy techniques are combined to achieve real-time and precise calibration detection of stone samples. Compared to traditional gout diagnosis methods, these techniques offer several advantages, including non-invasiveness, absence of labeling, simple sample processing, ease of operation, fast detection speed, and acquisition of rich information. The use of non-ionizing radiation also mitigates potential health risks. Compared with methods such as blood uric acid measurement, Raman and Terahertz detection can directly identify the composition of gout stones, without the need for indirect measurement methods to infer results, resulting in higher accuracy and reliability. Furthermore, the development of handheld Raman and Terahertz spectroscopic instruments offers the possibility of detecting samples in clinical settings, facilitating the early diagnosis and treatment of medical conditions such as gout. This study presents novel ideas and techniques for detecting metabolic diseases like gout and provides robust technical support to enable convenient clinical detection of diseases.

## 2 Materials and methods

### 2.1.1 Fourier transform infrared spectrometer (FTIR)

The Bruker IFS66v/s Fourier transform infrared spectrometer, produced by BRUKER in Germany, is a high-performance spectrometer used for infrared spectral analysis. The spectrometer includes an incoherent high-pressure mercury lamp, a far-infrared beam splitter, far-infrared focusing and collimating components, a nonthermal detector, an electric delay line, and sample, filter, and data acquisition systems. The Terahertz spectral region studied in this experiment effectively covers 30–680 cm^-1^, with a signal-to-noise ratio (SNR) better than 10000:1. When starting up, it is necessary to first turn on the water circulation and wait for half an hour for the system to stabilize before conducting experimental testing. In the experiment, parameters with a resolution of 4 cm^-1^, 128 scans, and a scanning speed of 5 kHz were used for spectral collection, resulting in a spectral frequency range of 0.9–20 THz. These parameters can ensure the high quality and accuracy of the experiment and are used for the detection and analysis of Terahertz spectroscopy.

### 2.1.2 Terahertz time domain spectroscopy system (THz-TDS)

**FIGURE 1:**
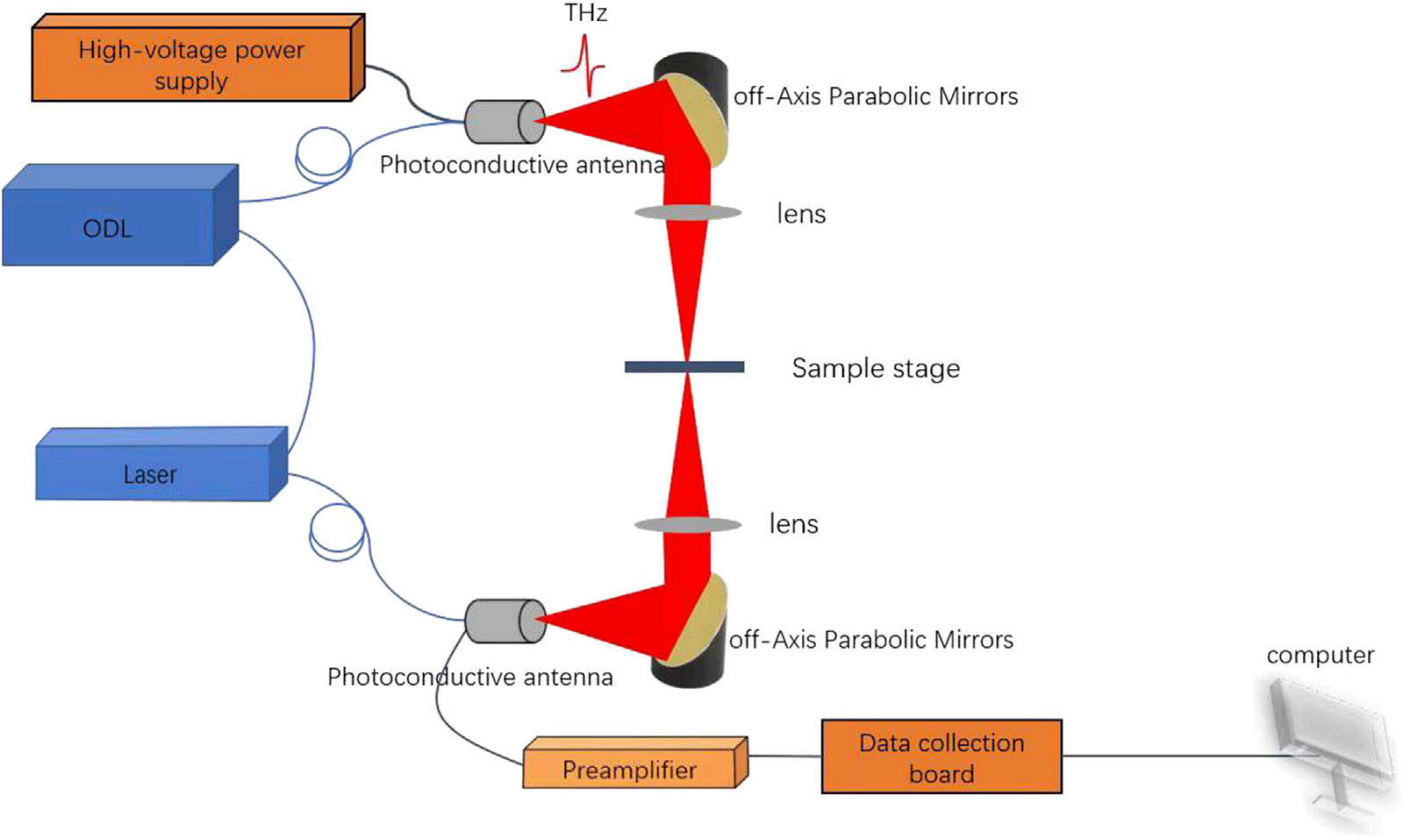
Composition of Terahertz time-domain spectroscopy system.

A Terahertz time-domain spectroscopy (THz-TDS) system designed by the Terahertz laboratory of the University of Shanghai for Science and Technology consists of a fiber femtosecond laser (1550 nm,<65 fs), optical delay line, collection board, amplifier, and Terahertz photoconductive transceiver antenna, the photoconductive antenna is fabricated using Indium Gallium Arsenide (InGaAs), as depicted in Figure 1. The main performance parameters of the system include a scanning speed of 0.1s per spectrum, an SNR exceeding 90dB, a spectrum width greater than 3THz, and a compact volume measuring 40 cm × 30 cm × 18 cm. The interior adopts a fully coupled optical path design with stable and reliable performance. In this experiment, the scanning range was 66ps, the scanning frequency was 512, and the scanning speed was 1440 mm/s. Firstly, air scanning is used as a reference signal, followed by testing the sample. Finally, the obtained data is processed and analyzed for spectral data.

#### 2.1.3 Laser confocal micro Raman spectrometer

The LabRAM HR Evolution, a micro Raman spectrometer produced by Horiba Scientific in France, is a powerful spectrometer that can be used for both micro and large sample measurements and has advanced 2D and 3D confocal imaging performance. The spectrometer is mainly composed of an open microscope/inverted microscope, high-precision three-dimensional platform, fiber optic probe, polarization attachment, transmission testing module, and other modules. It provides convenient analysis modules and can be widely used in Raman analysis, photoluminescence (PL), needle-enhanced Raman scattering (TERS), and other combined technologies. The LabRAM HR Evolution micro Raman spectrometer has high flexibility, can be extended to the full wavelength range (200 nm–2100 nm), and achieves automatic switching of all wavelengths. In this experiment, the analysis conditions for gout stones were as follows: laser light source wavelength 633nm, grating size 600gr/mm, measurement under a ×50 objective lens, exposure time 10s, scanning intensity 100%, and spectral collection range 200 cm^-1^–800 cm^-1^. The selection of these parameters can ensure high-quality spectral measurement and accurate analysis of the experiment, which is used for Raman spectroscopy analysis of gout stone samples.

### 2.2 Chemical reagents

Polyethylene was purchased from Sigma as a solid powder with particle diameters of 40–48 μm. The CAS number is 9002-88-4. Gout stones are a characteristic clinical manifestation of gout, which can easily deposit around the auricle and joints. But it is difficult to obtain. In this study, different types of urinary stones were selected to compare the analysis of gout stones and other stones, and the advantages of different detection schemes were analyzed. In the future, handheld portable comprehensive testing instruments will be developed to improve the efficiency and accuracy of testing.

All stone samples are sourced from the urinary calculi center of Shanghai Tenth People’s Hospital. The study protocol was approved by The Shanghai Tenth People’s Hospital’s Institutional Ethics Committee and was performed in accordance with the Helsinki Declaration.

### 2.3 Sample preparation and spectral collection

Before testing the sample of gout stone, it should be cleaned using distilled water and allowed to dry naturally. Subsequently, the sample should be placed in an oven and dried at a temperature of 40°C. To ensure proper particle size, the fully dried gout stone sample should be ground. This can be achieved by placing a 2 mm diameter steel ball along with the gout stone in a grinding tube and grinding them for 190 s using a grinder. The grinder parameters should be set to a frequency of 70 Hz and a time of 190 s. The grinding process aims to achieve a powder diameter of less than 2 μm. Different instruments used in the experiment require specific sample processing methods. For Terahertz spectral analysis, take 40 mg of gout stone and 10 mg of polyethylene (PE) and place them in a clean agate mortar. Thoroughly mix the materials using a grinding bowl and mortar, and grind them finely to obtain a mixture suitable for analysis. The mixed powder should then be placed into a mold. To obtain a uniform and particle-free thin sheet with a thickness of approximately 1 mm, a professional tablet press should be adjusted to apply a pressure of 3 tons. The pressing process should be continuous and uniform for 2 min, ensuring that the experimental quality loss is limited to within 1%. To prevent contamination, it is essential to clean the agate mortar and tablet mold thoroughly with pure non-woven fabric before preparing new samples each time. Additionally, it is necessary to suppress 40 mg of polyethylene as the background during the analysis. During the spectrum collection process for each sample, 3 points should be collected, and each point should be collected 3 times to obtain an average value.

### 2.4 Data processing and model establishment

Analyze the data using UnscramblerX 10.1 and Origin2018 to eliminate background interference, flatten the baseline, and perform correction processing on the data.

## 3 Results

### 3.1 Raman data processing and model establishment

**FIGURE 2:**
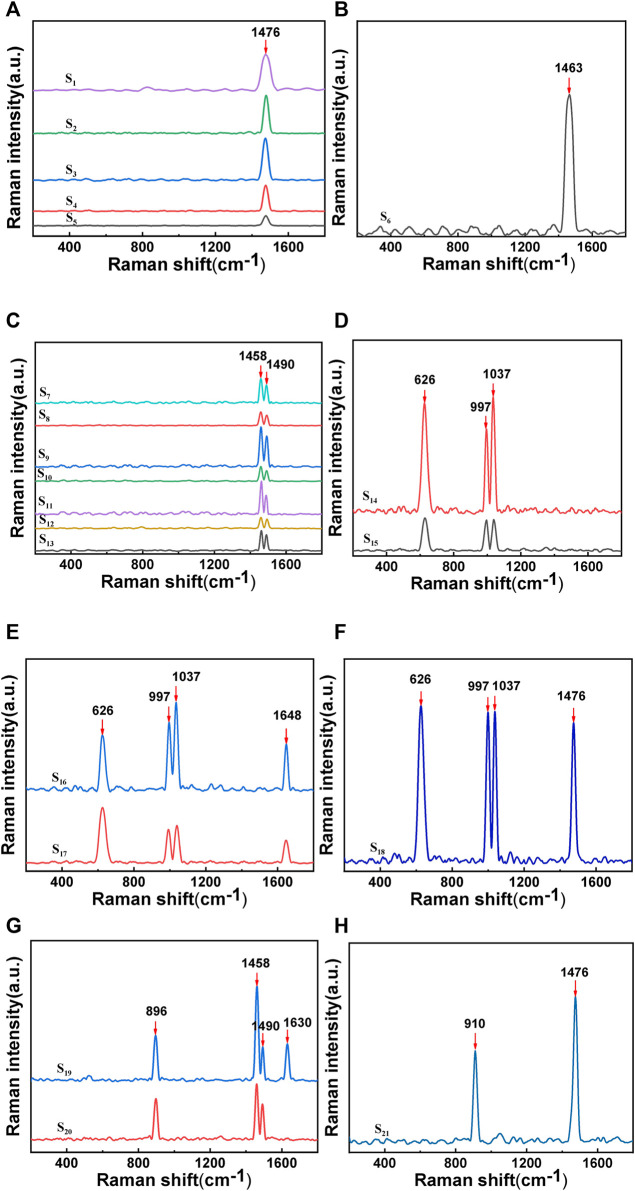
Raman spectra of stones:**(A)** and **(H)** show the Raman characteristic peak of calcium oxalate dihydrate;**(B)**, **(C)**, **(F)**, and **(G)** show the Raman characteristic peak of calcium oxalate monohydrate; **(D)**, **(E)**, and **(F)** show the Raman characteristic peak of uric acid.

A small amount of ground test sample was spread on a microscope slide and subjected to Raman spectroscopy. The tested samples were numbered sequentially as S_1_-S_21_, among which the Raman spectrum of uric acid (UA) is shown in Figure 2. The Raman peak at 1,476 cm^-1^ in (a) was due to the symmetric stretching vibration of C-O in calcium oxalate dihydrate (COD); the Raman peak at 1,463 cm^-1^ in (b) was due to the C=O vibration of calcium oxalate monohydrate (COM); the Raman peaks at 1,458 cm^-1^ and 1,490 cm^-1^ in (c) were both due to the C=O vibration of COM; the Raman peaks at 626 cm^-1^ in (d), (e), and (f) were due to the ring breathing vibration of the UA molecule; the peaks at 997 cm^-1^ and 1,037 cm^-1^ were due to the highly mixed vibration of UA; the Raman peak at 1,648 cm^-1^ in (e) was due to the C=O stretching vibration of UA; the Raman peak at 1,476 cm^-1^ in (f) was due to the symmetric stretching of C-O-O in COD; the Raman peak at 896 cm^-1^ in (g) was due to the C-C stretching of COM, and the Raman peak at 1,630 cm^-1^ in S_19_ was due to the symmetric stretching vibration of C-O in COM; the Raman peak at 910 cm^-1^ in (h) was due to the C-C shift stretching of COD (Sandra et al., 2022; Selvaraju et al., 2013; Cui et al., 2018).

In most Asian countries, the most common type of stone is calcium oxalate (75%–90%), specifically COM and COD, with UA (5%–20%) ranking as the second most common type (Fisang et al., 2015). Several studies have investigated the formation of calcium oxalate stones, revealing that COD crystals are the typical form of oxalate metabolism, while the presence of COM crystals may be an indication of increased risk for stone formation. Initially, calcium oxalate stones tend to crystallize in the form of COD rather than COM. This suggests that the transition from COD to COM occurs in the body, as COM is a relatively more stable form of the calcium oxalate compound (Costa-Bauzá et al., 2007). The application of Raman spectroscopy technology to identify and classify kidney stones can provide effective data for identifying and classifying stones at a molecular level without damaging the samples.

Among the samples of stones analyzed, it was found that the composition of stones in 21 patients was predominantly composed of calcium oxalate, accounting for 76.19%, with COM accounting for 47.62% and COD accounting for 28.57%. The proportion of UA was 23.81%, and mixed stones accounted for 4.76%, consistent with the literature (Fisang, Anding, Müller, Latz, and Laube, 2015). Raman spectroscopy measures the frequency shift and intensity changes of scattered light in the sample by irradiating it with a laser, providing vibration information of the sample and enabling the screening of different types of stones from mixed stones. Particularly for the identification of two types of gout stones, COM and COD, which have minor differences in their molecular structure and chemical composition, these differences can be captured and distinguished through the high resolution of Raman spectroscopy. This is important for the treatment of stones, as current clinical methods cannot clearly identify the two types. Research has demonstrated the feasibility of using Raman spectroscopy technology for identifying and classifying stones, thereby creating a novel approach for screening and diagnosing clinical stone types, with long-term research value. Although there are not many specimens, the proportion of pure uric acid stones is close to 25%. Early diagnosis and treatment of gout is very important.

### 3.2 Terahertz data processing and model establishment

#### 3.2.1 Fourier transform infrared spectrometer (FTIR)

FTIR and Raman spectroscopy are complementary technologies used for detecting basic molecular vibrations. However, FTIR is based on the absorption of light energy and the subsequent change in the dipole moment in the molecule, while Raman spectroscopy is generated due to the comprehensive scattering effect of light that occurs after the irradiation of monochromatic light, which causes the vibration of the change of polarizability in the molecule. Some molecules may have weaker vibrations in Raman spectroscopy but stronger vibrations in infrared spectroscopy (Paraskevaidi et al., 2021).

**FIGURE 3:**
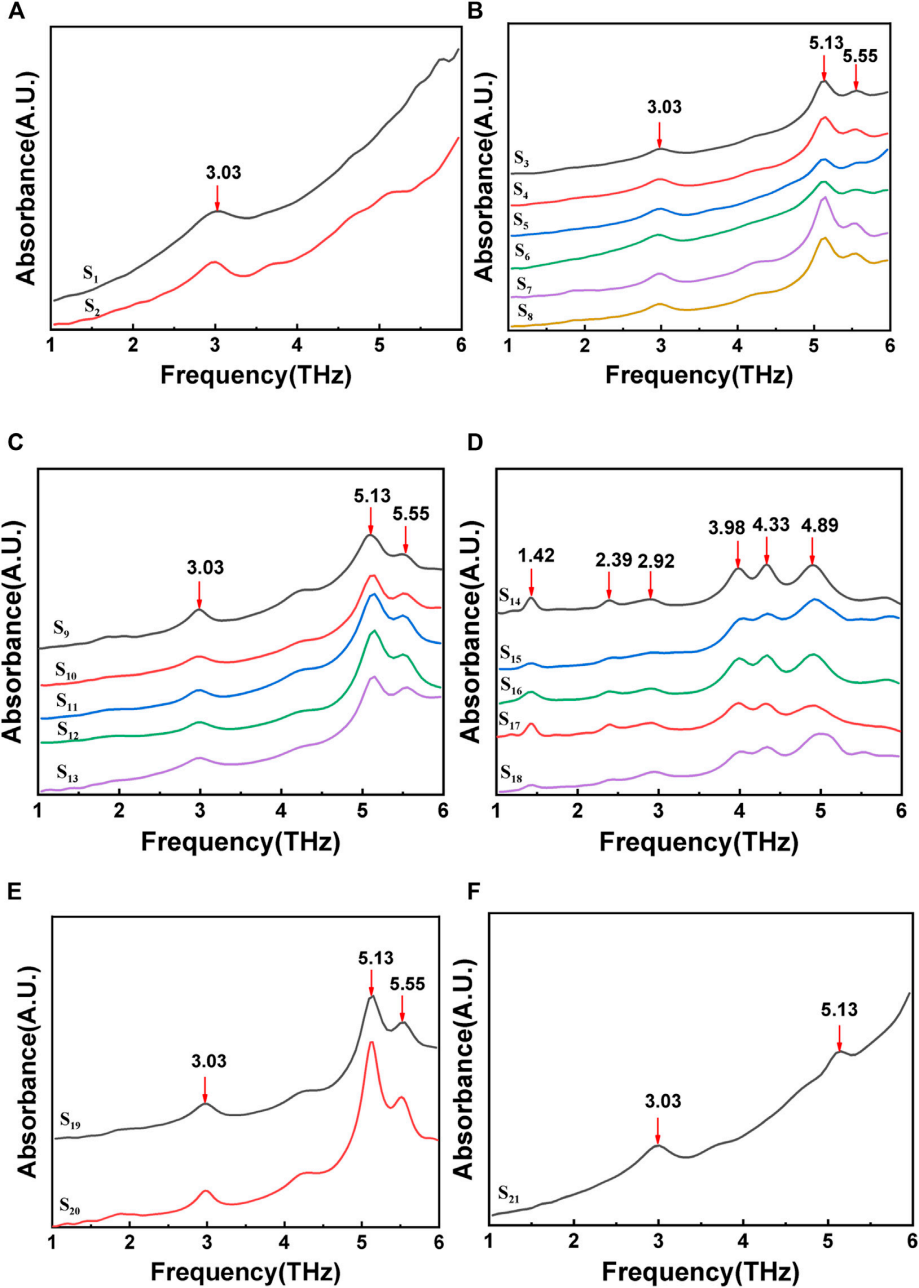
FTIR spectrum of gout stones:**(A)**, **(B)**, **(C)**, **(E)**,and **(F)** show the terahertz characteristic peak of calcium oxalate; **(D)** shows the terahertz characteristic peak of uric acid.

The compressed stone sample is placed under the laser, and the absorption spectrum in the 1-6 THz frequency band is measured using a Fourier transform infrared spectrometer. Figure 3 shows the FTIR spectra of gout stones. The characteristic absorption peak frequencies of 3.03 THz, 5.13 THz, and 5.55 THz for samples (a), (b), (c), (e), and (f) are compared to those in reference (Zhang, 2013; Wu et al., 2016). The stone component in these cases is CO, as shown in Figure 3D. Samples S_14_ to S_18_ exhibit significant Terahertz absorption peaks at 1.42 THz,2.39 THz, and 2.92 THz, indicating that the component of these stones is UA, based on a comparison of characteristic absorption peak frequencies in the literature (Zhang, 2013; Wu et al., 2016).

After conducting FTIR spectroscopy detection, it was determined that CO and UA are the primary components of stones in 21 patients, accounting for 76.19% and 23.81%, respectively. These findings are consistent with Raman measurements and indicate the feasibility of utilizing Terahertz spectroscopy for gout stone component detection. However, upon comparison with Raman results, it was discovered that the Terahertz detection could not differentiate between COM and COD, which may be due to the sample processing method. Terahertz detection operates on the stretching and rotational energies of functional groups in the low-frequency range. Prior to detection, the sample undergoes compression processing, which involves mixing the sample with polyethylene (PE) and pressing it into a sheet. This processing method may alter the structure and composition of the sample, consequently affecting the accuracy of Terahertz spectrum measurements.

#### 3.2.2 Terahertz time domain spectroscopy system (THz-TDS)

Fourier transform infrared spectroscopy and Raman spectroscopy are both analytical techniques used to detect information regarding molecular skeleton vibration and rotation, while X-ray diffraction is utilized to detect microstructural information of materials. In comparison, THz-TDS technology combines the benefits of the above three techniques, allowing for the precise analysis and identification of subtle changes in sample composition. THz-TDS technology captures low-frequency vibration information of molecular groups in the low-frequency Terahertz band with high time and frequency resolution, enabling fast and accurate analysis (Manceau et al., 2008). The data analysis and processing method of the THz-TDS system is consistent with that of the Fourier transform infrared spectrometer. Both methods use the fast Fourier transform (FFT) algorithm to convert time-domain information into frequency-domain information.

**FIGURE 4:**
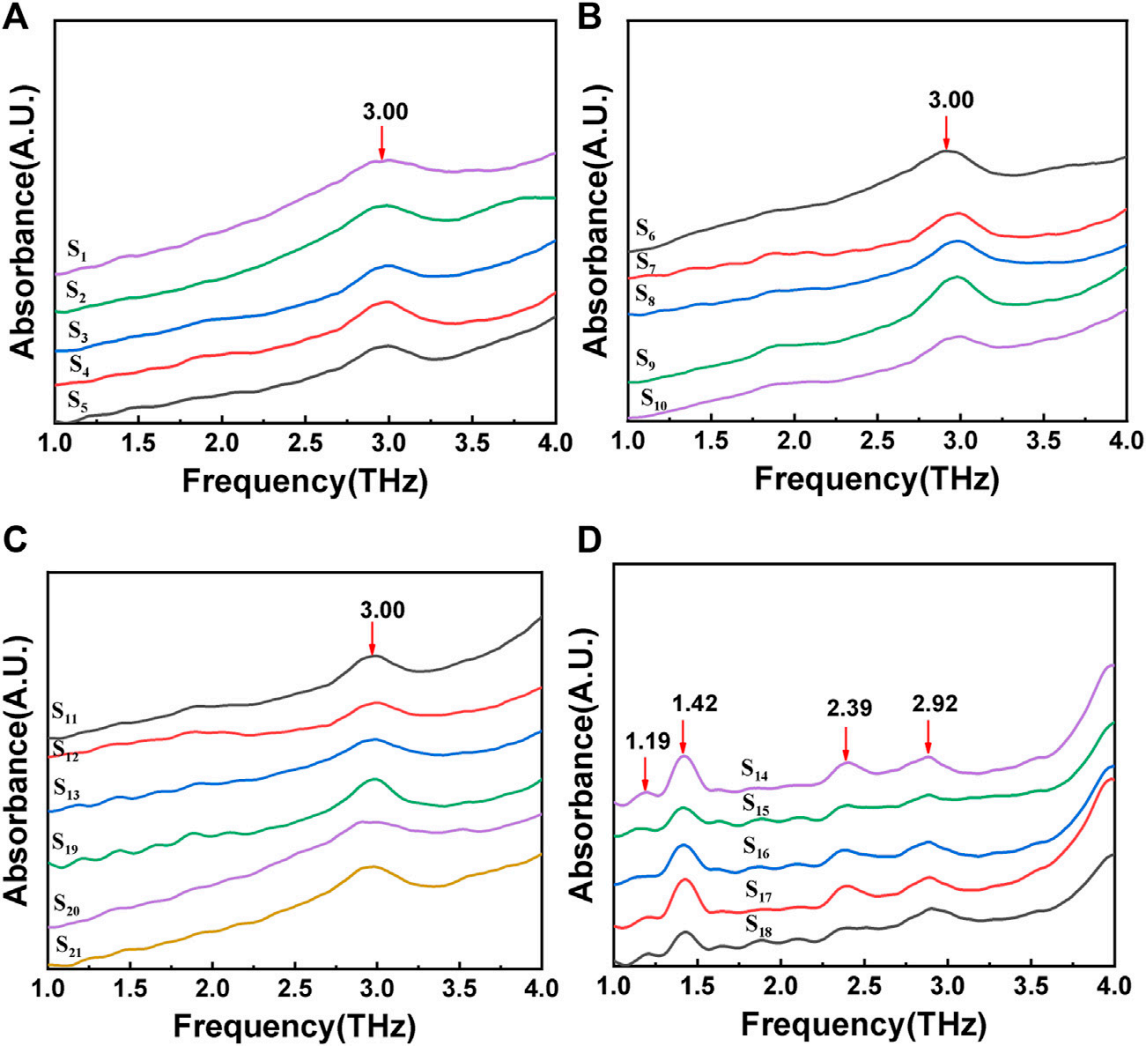
THz-TDS spectrum of gout stones:**(A)**, **(B)**, and **(C)** show the terahertz characteristic peak of calcium oxalate; **(D)** show the terahertz characteristic peak of uric acid.

The absorption spectrum of gout stones in the 1-4 THz frequency band was measured using THz-TDS by placing the compressed sample under a self-built laser. Figure 4 displays the resulting spectrum, revealing several Terahertz absorption peaks. A comparison of the characteristic absorption peak frequencies from the literature (Zhang, 2013; Wu et al., 2016) at 3.00 THz (a), (b), and (c) indicates that the gout stone component is CO. The Terahertz characteristic peak of CO was found to be 3.00 THz, as shown in Figure 4, which agrees with the FTIR-measured peak of calcium oxalate in Figure 3, indicating the accuracy of the measured data and the feasibility of instrument validation for gout composition. Furthermore, the analysis of the THz-TDS spectrum revealed additional Terahertz absorption peaks at 1.19, 1.42, 2.39, and 2.92 THz in the gout stone, which are characteristic peaks of UA (Chen et al., 2020). Thus, the main component of the gout stone was identified as UA. This additional peak at 1.19 THz, not detected by FTIR, suggests that the self-built THz-TDS system can achieve high-frequency resolution in detecting low-frequency vibration information of molecules in the low-frequency Terahertz band, which is crucial for the development of handheld portable Terahertz detection instruments. However, there are certain differences in the spectra of CO samples due to the lower signal-to-noise ratio of FTIR Terahertz detection compared to THz-TDS. The unstable laser light source or subtle noise signals can impact the measurement. Additionally, the wide spectrum of FTIR measurement leads to a larger frequency interval between each two scanning points within similar scanning times, which can easily affect the measurement results, especially for low Terahertz test results. Therefore, an error of 0.03 THz in the data analysis process can be considered under allowable error conditions.

Moreover, in Figure 4C, some samples exhibited relatively consistent waveforms before 3.00 THz.To investigate this further, multiple samples were measured, and the same sample was measured multiple times in the experiment. The resulting measurement results were compared, revealing similar peak positions at the same frequency that remained relatively stable within the measurement range. Therefore, it is likely that these peak positions represent the natural frequencies of the instrument rather than the characteristic frequency analysis of the samples.

## 4 Discussion

Gout is a metabolic disorder characterized by abnormal metabolism of uric acid, which results in increased blood uric acid levels and the formation of urate salts in joints and soft tissues, leading to the formation of gout stones. Gout stones can cause serious health problems such as arthritis, joint pain, and kidney disease. Current diagnostic methods for gout mainly rely on blood uric acid level tests, arthritis symptoms, and X-rays, but these have certain limitations in the early diagnosis and treatment of gout stones. In this study, 21 gout stone samples were analyzed using three different instruments: laser confocal micro raman spectrometer, FTIR, and THz-TDS. The results indicate that the main components of gout stones can be determined by their characteristic absorption peaks with relatively small errors. Different detection method pairs are shown in Table 2. Compared with commonly used medical examination methods, THz-TDS detection can non-destructively detect the internal structure and physical properties of samples, which fills the gap in the mechanism of low-energy band interactions with substances that cannot be covered by the stretching and rotating energy of functional groups in the infrared range, such as hydrogen bonds, intermolecular van der Waals forces, and detecting small changes and defects. Raman spectroscopy technology can provide more detailed and accurate chemical information, analyze the molecular composition and structure of samples, and can be used for chemical analysis and material research. In terms of gout stone detection, Raman spectroscopy has high sensitivity and resolution, which means that even at low concentrations of gout stone samples, detailed identification of different chemical components and structures of gout stone samples can be carried out. A deep understanding of the characteristics and properties of gout stones is helpful for early diagnosis and treatment. Therefore, in clinical medicine, Terahertz and Raman spectroscopy techniques can complement each other to form more comprehensive analysis results.

**TABLE 2:** Comparison of optical testing methods.

Test method	Advantages	Application scope	Reference
Laser Confocal Micro Raman Spectrometer	Non-invasive Real-time	It has molecular specificity and provides high-resolution information at the cellular level for studying molecular composition and microstructure	Movasaghi et al. (2007)
Fourier Transform Infrared Spectrometer (FTIR)	High resolution	A wider spectral range is used to analyze more types of molecules, which can be used to study histology and chemical composition	Jamrógiewicz (2012)
Terahertz Time Domain Spectroscopy System (THz-TDS)	Diversity Information	Extremely high Spectral resolution and time resolution can be used to study physical properties, dynamics, and thermodynamic properties	Castro-Camus et al. (2021)

From a clinical perspective, Terahertz and Raman spectroscopy are non-ionizing radiation detection techniques that do not pose any health risks to the human body, making them suitable for long-term disease tracking. In contrast, other imaging methods such as X-ray and CT scanning generate radiation and may cause potential health hazards (Esmonde-White et al., 2017; Demir and Severcan, 2016; Ajito, 2015; Kawase et al., 2003). Moreover, each component in gout stones has a distinct Raman or Terahertz spectrum, which can be used for spectral analysis. The unique “fingerprint spectrum” is used to conduct spectral tests on samples with known components to establish a corresponding spectral database. By comparing the spectral spectra in the database, it is possible to quickly identify and classify samples of unknown components, achieve automated analysis, improve analysis speed and efficiency, and reduce dependence on experimental personnel. Establishing a sample database not only provides more comprehensive data support for research but also promotes application and accelerates the development and popularization of technology. Raman and Terahertz spectroscopy technology is user-friendly and simple to use. Combining Raman-Terahertz spectroscopy technology with computer simulation methods has enabled quick and accurate detection of defects in organic and inorganic crystals and has provided detailed atomic structure and electronic property information (Nishizawa et al., 2006). Handheld devices are also available to detect samples with the same accuracy as traditional laboratory analysis methods (Vargas Jentzsch et al., 2016; Molter et al., 2011; Molter et al., 2011). The development of handheld portable integrated testing instruments has emerged as a solution to circumvent the requirements of specialized laboratory conditions and laborious preprocessing procedures. It effectively mitigates the cost and time inefficiencies associated with various laboratory analysis methods, while streamlining the diagnostic process. Early identification of gout stone types and prompt initiation of treatment facilitate inflammation reduction and prevent complications. Therefore, the establishment of a Raman-Terahertz spectrum library in clinical medicine assumes paramount importance. Through this study, we aim to construct a comprehensive stone composition database for *in vitro* detection of urinary system stones. In future endeavors, our objective is to achieve early detection and diagnosis of urinary system stones and perform gout stone composition analysis using portable instruments, facilitated by urine centrifugation sediment and freeze-drying grinding of solid residue. This approach promises precise and comprehensive diagnoses, along with the exploration of novel clinical applications, thereby enhancing the provision of superior medical services to patients.

## Data Availability

The raw data supporting the conclusions of this article will be made available by the authors, without undue reservation.
